# Preparation and characterization of 100% bio-based polylactic acid/palmitic acid microcapsules for thermal energy storage

**DOI:** 10.1007/s40243-017-0098-0

**Published:** 2017-06-30

**Authors:** Maryam Fashandi, Siu N. Leung

**Affiliations:** Department of Mechanical Engineering, Lassonde School of Engineering, York University, Toronto, ON M3J 1P3 Canada

**Keywords:** Bio-based microPCM, Encapsulation, Palmitic acid, Polylactic acid, Solvent evaporation

## Abstract

Phase change materials (PCM) have gained extensive attention in thermal energy storage applications. In this work, microencapsulation of vegetable-derived palmitic acid (PA) in bio-based polylactic acid (PLA) shell by solvent evaporation and oil-in-water emulsification was investigated. Fourier transform infrared spectroscopy and scanning electron microscopy were conducted to confirm the successful encapsulation of PA in PLA shells. Differential scanning calorimetry was performed to evaluate the thermal properties, thermal reliability, and core content of the fabricated PCM microcapsules (microPCM). Through a series of parametric studies, the effects of PCM and solvent content, oil phase-to-aqueous phase ratio, as well as surfactant type and content on the morphology, particle size, and thermal properties of the PCM microcapsules were investigated. Experimental results showed that PVA was a superior emulsifier to SDS in the emulsion systems being studied. There also existed an optimal PVA concentration to reduce the average size of microPCM. When the PVA concentration was above this optimal level, the emulsifier molecules tend to form micelles among themselves. This led to the adhesion of tiny microspheres on the surface of microPCM as well as larger microPCM. In short, this work has demonstrated the possibility of using the solvent evaporation method to fabricate 100% bio-based PCM-polymer microcapsules for thermal energy storage applications.

## Introduction

Around 40% of the world’s energy consumption, and one-third of the global greenhouse gas emissions are related to the building sector [1]. Greenhouse gases are known to be the main reason of global warming and scientists have predicted that by the end of twenty-first century, the average temperature of the Earth can rise by up to 7 °C [2]. The reduction in energy consumption and an improved energy preservation system represent reasonable ways on suppressing the greenhouse gases emission, and thereby reducing the rate of global warming. In this context, phase change materials (PCM) seem to be a possible solution that can help reduce the need to non-renewable fossil fuels. During the phase transition (i.e., solid–liquid, solid–gas or liquid–gas), the energy will be stored in or released from PCM [3]. When PCM undergo isothermal melting, sublimation or vaporization, energy is absorbed from the surrounding and saved in the material. In contrast, when PCM solidify or condense, energy is released. Due to their wide range of melting points, different PCM are suitable for thermal energy storage applications under diverse environments. Although PCM undergoing liquid–gas transition have higher heat storage capacity than those undergoing solid–liquid transition, the latter have the higher practicality because of their smaller volume changes in comparison to PCM that undergo liquid–gas transition [4, 5].

PCM can be classified as organic, inorganic. and eutectics. The amount of thermal energy that can be stored in inorganic PCM, like salts, salt hydrates, metals and alloys, are up to two times the amount that can be stored in organic ones. They are also nonflammable, cheaper and possess a better thermal conductivity in comparison to organic PCM. However, their corrosiveness as well as sub-cooling and super-cooling behaviors have limited their applications [6, 7]. Organic PCM can be subdivided into paraffinic and non-paraffinic materials. Paraffinic petroleum-based PCM are the most commonly used PCM. They possess respectable amount of latent heat. They also have limited super-cooling, low reactivity, good thermal and mechanical stability, low vapor pressure, as well as self-nucleating ability. However, their drawbacks include low thermal conductivity, flammability, and being more expensive than inorganic PCM [4, 8, 9]. Non-paraffinic PCM are mostly bio-derived from vegetable oil (e.g., soybean oil, coconut oil, palm oil, etc). Furthermore, fatty acids, esters, alcohols and glycols are some well-known examples of this subcategory of PCM [10]. By covering a wide range of melting temperatures, these bio-based PCM are appropriate candidates for thermal energy storage applications in different environments. Similar to paraffinic PCM, they have high latent heat, low vapor pressure, good chemical and thermal stability, fast self-nucleating behavior, and high abundance. Due to their fully hydrogenated structure, they can undergo thousands of thermal cycles without oxidation. With lower flammability and cheaper price than paraffinic PCM while possessing all the positive properties of them [11, 12], bio-based PCM represent a great choice for thermal energy storage purposes.

When PCM undergo phase transition from their solid state to liquid state, they start to flow. In this context, encapsulation is a widely used technique to prevent PCM from migrating and reacting with their environment. If the encapsulation results in PCM capsules in micron-scale [i.e., denoted as microencapsulated phase change material (microPCM)], it would also increase their surface-to-volume ratio, thereby promoting heat transfer into and from their cores. It must be noted that a proper encapsulating material should meet the criteria under which the microPCM operates, such as compatibility, strength and flexibility, needed in specific applications. On the one hand, the encapsulating shells should protect PCM from any damage or leakage during operation. On the other hand, they should be compatible with the operating environment while they are in direct contact with food, medicine, people, etc [13].

Two main methods for encapsulating PCM are macroencapsulation and microencapsulation. They are different in terms of the final capsule sizes. Microencapsulation is defined as wrapping any material in a capsule with its diameter between 1 and 1000 µm. Beyond this range, the capsules are called macrocapsules [4]. Comparing the two encapsulation approaches, microencapsulation results in greater surface area-to-volume ratio to enhance the heat transfer performance, improved durability, and suppressed supercooling [14]. Extensive researches were conducted to encapsulate various organic and inorganic PCMs by a wide range of polymers, such as polystyrene [15], poly(methyl methacrylate-*co*-divinylbenzene) [16], phenolic resin [17], vinyl trimethoxysilane [18], and urea–formaldehyde resin [19]. Furthermore, researchers investigated different fabrication techniques to encapsulate PCM. These include in situ polymerization [16, 20] interfacial polymerization [14], emulsion polymerization [21], condensation polymerization [22], and solvent evaporation [23]. Depending on their melting points, encapsulated PCM can be used in many different areas, including food and pharmaceutical preservation, blood transport, solar power plants, electronic devices, space equipment’s, textile, sportswear, hot and cold therapies, building materials, etc. [24, 25].

While microPCM represent a unique means to promote substantial energy conservation through their passive thermal regulating and thermal energy storage capabilities, it is possible to promote further their environmental sustainability if both the microscopic shells and PCM cores are bio-based. To the best knowledge of the authors, the design and fabrication of 100% bio-based microPCM have yet been reported. As a result, the goals of this study are to develop a fabrication strategy to produce 100% bio-based microPCM as well as to investigate the effects of different processing parameters on their morphological and thermal properties. In this context, palmitic acid (PA) and PLA shell were used herein as case examples of the PCM core and the protective shell, respectively. PA is a fatty acid with negligible supercooling and volume change during phase transition. It also has high latent heat of fusion, superior thermal stability, and no toxicity. In order to enhance its thermal conductivity, researchers have dispersed graphite [26] and carbon nanotubes [27] in them. Furthermore, PA has also been added as a filler to SiO_2_ [28],TiO_2_ [29] and polypyrrole [30], as well as an eutectic PCM with stearic acid [31], capric acid [32], myristic acid, and *n*-octadecane [33]. PLA is a biodegradable and bio-based polymer with its physical and mechanical properties comparable to conventional commodity polymers such as polystyrene. This has made PLA an attractive option to be used as the shells of microPCM to promote their environmental sustainability [34]. Although synthesizing this bio-based polymer through various methods, such as ring opening polymerization, polycondensation, enzymatic polymerization, etc., is quite complicated, their production needs 25–55% less energy in comparison to petroleum-based polymers. Moreover, this FDA-approved polymer can be degraded into non-toxic materials, which makes it a great alternative to conventional petroleum-based polymers in different applications [35, 36].

## Experimental

### Materials

PLA (Ingeo 8052D NatureWorks LLC) was used as the polymeric shell of the microPCM. PA (Acros organics) was used as the PCM core. 87–89% hydrolyzed polyvinyl alcohol (PVA, Sigma Aldrich) with an average molecular weight of 146,000–186,000 g mol^−1^ and sodium dodecyl sulfate (SDS, Sigma Aldrich, ReagentPlus grade) were used as emulsifiers. Dichloromethane (DCM, Caledon Laboratory Chemical), with the density of 1320 kg m^−3^, was the organic solvent for the preparation of the oil phase. Deionized water was the aqueous medium. All materials and chemicals were used as received.

### Preparation of PLA–PA microPCM

Microencapsulation of PA core in PLA shell was conducted by the solvent evaporation method accompanied by oil-in-water emulsification [37]. This method has been widely used in the pharmaceutical industry to encapsulate different medicines with bio-based and biodegradable polymers such as PLA and poly(lactic-*co*-glycolic acid) (PLGA) due to its simplicity and ability to produce repeatable results [38]. In this method, pre-synthesized PLA is used, and this eliminates the complicated PLA polymerization step. Unlike emulsion polymerization, no monomer, initiator or catalyst is used. Hence, it allows the fabrication of microspheres with high purity [39]. In general, the fabrication process involves four main steps: (1) dissolution of PA and PLA in DCM; (2) emulsification of this organic phase (i.e., dispersed phase), in a continuous aqueous phase of deionized water containing PVA; (3) extraction and evaporation of solvent from the dispersed phase, which transform the dispersed phase into solid microspheres (i.e., microPCM); and (4) recovery and drying of microPCM to eliminate residual solvent and emulsifier.

For the oil phase, 1.2 g of PLA and different amounts of PA (i.e. 0.4, 0.6, or 0.8 g) were added to DCM. The mixture was stirred for 2 h at 36 °C, which was lower than the boiling point of DCM (i.e., 40 °C), to obtain a uniform PLA–PA solution. For the aqueous phase with PVA as the emulsifier, a uniform PVA solution was prepared by dissolving PVA in deionized water. The solution was cured for 30 min at room temperature to swell PVA. After that, it was heated to 80 °C for 3 h to ensure complete dissolution of PVA. For the aqueous phase with SDS as the emulsifier, a desired amount of SDS was dissolved in deionized water at room temperature. Consequently, the oil-in-water emulsion was prepared by adding 5 or 10 g of oil phase solution to 60 g of aqueous solution. The oil–water system was first stirred by a magnetic stirrer at 200 rpm for three hours at room temperature. The emulsion was then sonicated at an amplitude of 50% level for 5 min using a sonicator probe (QSonica Q700). In the next step, DCM was evaporated by elevating the emulsion temperature to 70 °C while the emulsion was continuously stirred at 200 rpm for 1 h. The remaining solution was kept at room temperature for 48 h to precipitate the fabricated microPCM. Eventually, microPCM were repeatedly washed with deionized water at 50 °C and filtrated to remove the PVA residues. The washed microPCM were dried in a vacuum oven at 50 °C for 12 h. Table 1 summarizes the material compositions used to fabricate the PLA–PA microPCM in this work. PCM0.6 was denoted as the base case for comparison purpose.

**Table 1:** Conditions for the preparation of PLA–PA microPCM

Sample	Oil phase			Aqueous phase			Oil-in-water ratio
	PLA (g)	PA (g)	DCM (mL)	PVA (g)	SDS (g)	DI water (g)	
PCM0.4	1.2	0.4	29	5	–	95	1:12
PCM0.6	1.2	0.6	29	5	–	95	1:12
PCM0.8	1.2	0.8	29	5	–	95	1:12
PCM0.6_DCM0.5_	1.2	0.6	14.5	5	–	95	1:12
PCM0.6_PVA2_	1.2	0.6	14.5	2	–	98	1:12
PCM0.6_PVA3_	1.2	0.6	14.5	3	–	97	1:12
PCM0.6_PVA4_	1.2	0.6	14.5	4	–	96	1:12
PCM0.6_O/W×2_	1.2	0.6	14.5	2	–	98	1:6
PCM0.6_PVA2-SDS2_	1.2	0.6	14.5	2	2	96	1:12
PCM0.6_SDS0.5_	1.2	0.6	14.5	–	0.5	99.5	1:12
PCM0.6_SDS1_	1.2	0.6	14.5	–	1	99	1:12
PCM0.6_SDS2_	1.2	0.6	14.5	–	2	98	1:12
PCM0.6_SDS3_	1.2	0.6	14.5	–	3	97	1:12
PCM0.6_SDS4_	1.2	0.6	14.5	–	4	96	1:12

### Characterization of PLA–PA microPCM

The chemical structures of microPCM and each of its components (i.e., PLA and PA) were analyzed using Fourier transform infrared (FTIR) spectroscopy (Bruker Alpha-P FT-IR Spectrophotometer). The spectra were collected by averaging signals from 32 scans at a resolution of 4 cm^−1^ in the range of 400–4000 cm^−1^.

Scanning electron microscopy (SEM) (FEI Company, Quanta 3D FEG) was used to observe the morphologies (i.e., surface features and sphericity) and sizes of microPCM. The fabricated microPCM were sputter coated with gold (Denton Vacuum, Desk V Sputter Coater) before the observation. The particle sizes were obtained by analyzing the SEM micrographs using ImageJ (NIH Image). The interior morphology of microPCM was exposed by microtoming microcapsules using a diamond knife.

The enthalpy of fusion and the melting point of microPCM were determined by a differential scanning calorimetry (DSC) (TA Instrument, DSC Q20). These measurements were performed in the temperature range from 40 to 90 °C at a heating rate of 10 °C min^−1^. In order to determine the thermal stability of the microPCM, the enthalpy of fusion was analyzed after samples [i.e., the base case (PCM0.6)] were subjected to 50 thermal cycles at the same temperature range and heating rate.

## Results and discussion

### Chemical structures of PLA–PA microPCM

**Fig. 1:**
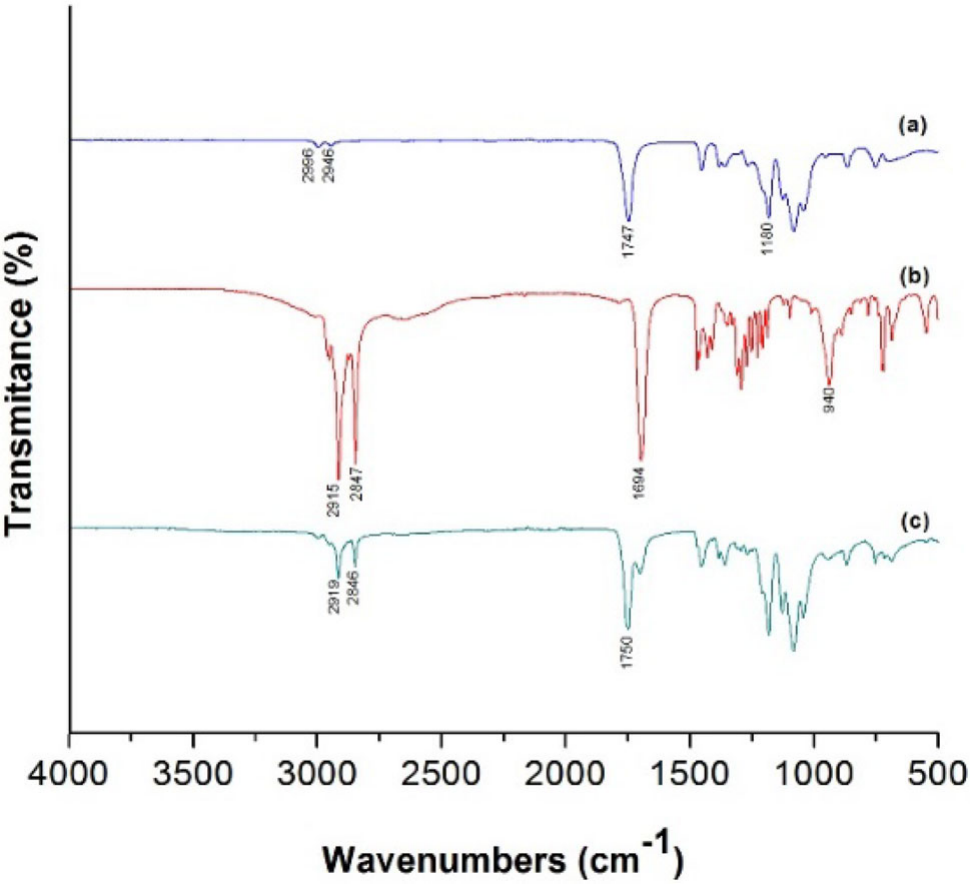
FTIR spectra of: **a** PLA; **b** PA; and **c** PLA–PA microcapsules

The FTIR spectra of PLA, PA, and PLA–PA microPCM (i.e., PCM0.4) were illustrated in Fig. 1. From curve (a), it can be observed that PLA had obvious absorption peaks at 2996 and 2946 cm^−1^, which corresponded to stretching vibration of CH_3_ bond in the molecular structure. The sharp peaks around 1747 and 1180 cm^−1^ were related to the stretching vibration of C=O and C–O–C bond.

Curve (b) shows the FTIR spectrum of PA. The sharp peak at 2847 cm^−1^ was resulted from the stretching vibration of CH_2_ groups and the peak at 2915 cm^−1^ was caused by the stretching vibration of CH_3_ group in the PA structure. The absorption band from 2500 to 3300 cm^−1^ belongs to the stretching vibration of –OH group and the peaks at 1694 and 940 cm^−1^ were related to the stretching of C=O and the out of plane bending of –OH, respectively. [28, 40].

The FTIR spectrum of the PLA–PA microPCM is shown in curve (c). The characteristic peak at 2846 cm^−1^, attributed to CH_2_ group, belonged to the PA. The absorption peak at 2919 cm^−1^ was related to CH_3_ bond, which was related to both the PLA shell and the PA core. The sharp peak at 1750 cm^−1^ was attributed to C=O group, which could also be found in both materials. However, the significantly lower transmittance in the peak related to the CH_2_ group comparing to the FTIR spectrum of pure PA revealed that both PLA and PA existed in the microPCM. It can be seen that the absorption peaks of PLA–PA microPCM were consistent with those of pure PA and PLA. Therefore, it can be concluded that PA was well encapsulated by PLA resin and no chemical interaction occurred between the core and the shell materials.

### Thermal properties and thermal reliability of PLA–PA microPCM

PLA–PA microPCM fabricated by using different material compositions were analyzed by DSC to compare their performances and operating temperatures for thermal energy storage. Neat PA’s melting point and latent heat of fusion were 62.8 °C and 167.3 J/g, respectively. The latent heat of fusion of microPCM can be determined by integrating the area under the endothermic peak in a DSC thermogram. By comparing this with the latent heat of fusion of pure PA using Eq. (1), the core content of PLA–PA microPCM can be calculated.

1 $$Core \, Content = \frac{\Delta H}{{\Delta H_{PCM} }} \times 100\% ,$$

where Δ*H* and Δ*H*_PCM_ are the latent heat of fusion of microPCM and that of PA, respectively.

The melting temperatures, the enthalpies of fusion, and the core contents of different microPCM samples are summarized in Table 2. The results show that the melting temperatures for all microPCM samples were virtually unchanged, with a mean temperature and standard deviation of 62.2 and 0.2 °C, respectively. In contrast, the core content was strongly dependent on the PA content as well as the type of emulsifier used. For microPCM prepared by using PVA as the emulsifier, as the PA loading increased from 0.4 to 0.8 g, the core content increased from 24.3 to 41.9%. Nevertheless, changing the DCM content, the PVA content, or the oil-to-water ratio only had minor effects on the core contents of PLA–PA microPCM.

When comparing the two emulsifiers (i.e., PVA and SDS), experimental results reveal that PVA was significantly more effective than SDS for the microencapsulation of PA cores by PLA shells. While all microPCM prepared by using 0.6 g of PA and various amounts of PVA resulted in similar core contents, the endothermic peak of PA was either absent or suppressed in the DSC thermograms of microPCM prepared by using SDS as the lone emulsifier or together with PVA. The hydrophile-lyophile balance (HLB) of SDS is higher than that of PVA [41]. While PVA had a good solubilizing ability to prepare micro-emulsion of oil phase in the aqueous phase, the excessively high HLB value of SDS, together with its high PA solubility, had made it act as a perfect solubilizing agent to dissolve PA completely in the aqueous phase. As a result, the PA core materials were removed with the aqueous phase in the filtration and washing steps. Although lowering the SDS content to 1.0 wt% or below helped to sustain some PA cores in the microPCM, the resultant core contents were significantly lower than those of microPCM prepared by using PVA as the emulsifier. As a result, SDS was an inappropriate surfactant for this oil-in-water emulsion system [42] (Table 2).

**Table 2:** Thermal properties of PLA–PA microPCM

Sample	Melting point (°C)	Enthalpy of fusion (J/g)	Core content (%)
PCM0.4	61.9	40.7	24.3
PCM0.6	62.3	59.9	35.8
PCM0.8	62.1	70.1	41.9
PCM0.6_DCM0.5_	62.4	55.1	32.9
PCM0.6_PVA2_	62.5	52.8	31.5
PCM0.6_PVA3_	62.2	54.3	32.4
PCM0.6_PVA4_	62.3	51.9	31.0
PCM0.6_O/W×2_	62.0	62.2	37.1
PCM0.6_PVA2-SDS2_	–	–	–
PCM0.6_SDS0.5_	61.9	33.2	19.8
PCM0.6_SDS1_	62.4	12.0	7.2
PCM0.6_SDS2_	–	–	–
PCM0.6_SDS3_	–	–	–
PCM0.6_SDS4_	–	–	–

The microPCM must maintain their performances in practice after long-term uses. Therefore, it is important that they have insignificant change in thermal properties after repeated thermal cycles. In this context, a thermal cycling test was conducted on the microPCM prepared under the base case conditions (i.e., PCM0.6) to determine the thermal reliability of PLA–PA microPCM. After undergoing 50 thermal cycles over a temperature range of 40–90 °C, the enthalpy of fusion decreased by only 1.0 to 58.9 J/g. This result indicates that there was no chemical degradation in the fabricated microPCM during thermal cycling and provides evidence to confirm the microPCM were stable chemically after repeated thermal cycling.

### Morphology and size distribution of PLA–PA MicroPCM

**Fig. 2:**
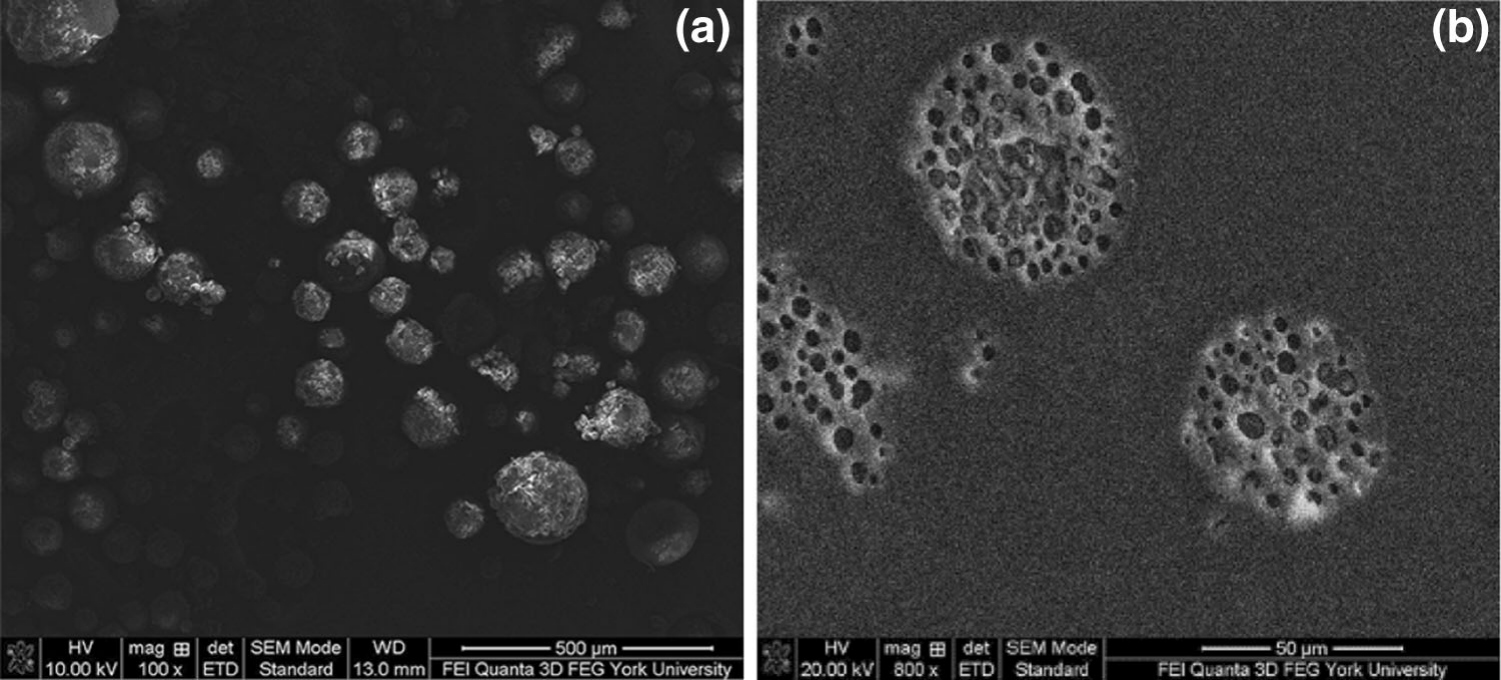
SEM micrographs of PLA–PA microPCM (i.e., PCM0.6): **a** a batch of microPCM; and **b** cross-sections of individual microPCM

Figure 2a and b show the SEM micrographs of a batch of the base case PLA–PA microPCM (i.e., PCM0.6) and the cross-sections of individual microcapsules, respectively. Figure 2a reveals that the fabricated microPCM were generally spherical but with a wide range of size distribution and relatively uniform exterior characteristics. This suggests successful microencapsulation of PA cores by PLA shells. The interior features of microPCM were revealed by performing SEM on the microtomed samples. Figure 2b illustrates that individual microPCM possessed multi-core morphologies, which was consistent with the general expectation for a microencapsulation process involving an emulsification step [43].

In this section, the effects of (i) PCM core content; (ii) oil and aqueous media; and (iii) emulsifier type and content on microPCM’s morphology and size distribution are discussed. The average sizes of microPCM prepared by different material compositions were analyzed using SEM and plotted, with the error bars representing the standard deviations of the microPCM’s sizes.

**Fig. 3:**
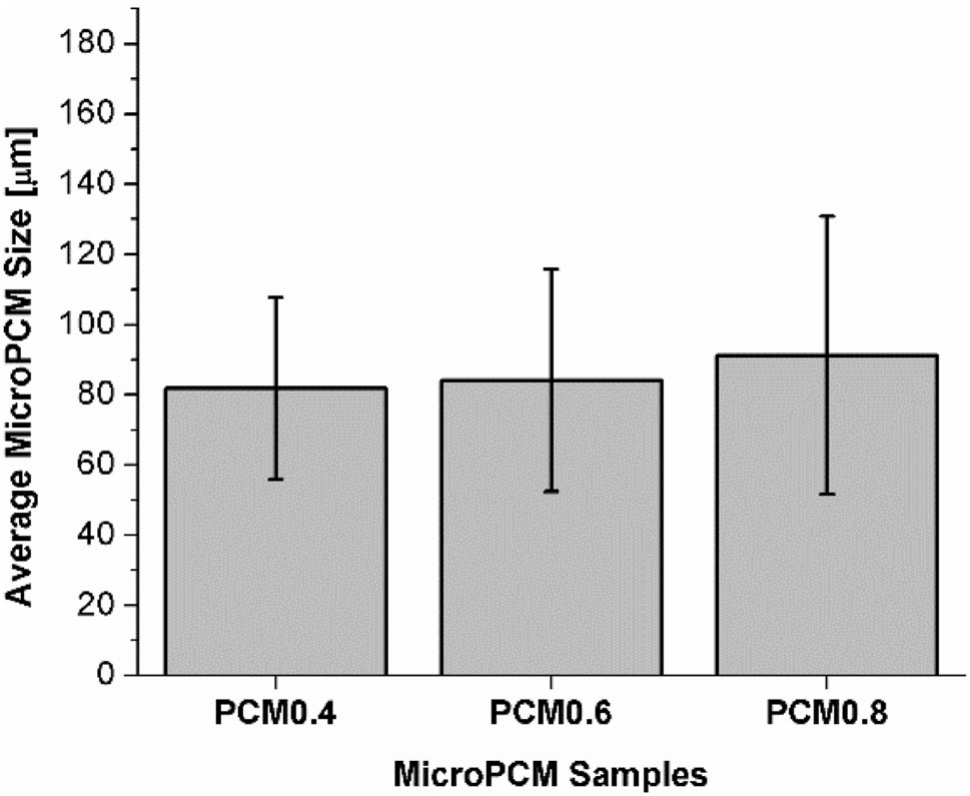
Effect of PA content on microPCM’s sizes

Figure 3 plots the average microPCM sizes and the standard deviations for samples consisted of different PA contents. All samples were fabricated at the same processing conditions as the base case. The results reveal that increasing PA contents would slightly increase microPCM’s average sizes as well as the degrees of size variation. Research related to the encapsulation of drug by the solvent evaporation approach reported that increasing the viscosity of the dispersed phase in the emulsion would increase the size of the microcapsule. This relationship was expressed as an empirical model in Eq. (2) [37]. When preparing PLA–PA microPCM, increasing the PA content would increase the viscosity of the dispersed phase, and thereby resulted in larger microPCM sizes.

2 $$d = A\left( {\frac{{\mu_{\text{d}} }}{{\mu_{\text{c}} }}} \right)^{0.25} \times 100\% .$$

where *d* is the average diameter of the microspheres, *A* is a coefficient dependent on process conditions, *µ*_d_ is the viscosity of the dispersed (oil) phase, and *µ*_c_ is the viscosity of the continuous (aqueous) phase.

**Fig. 4:**
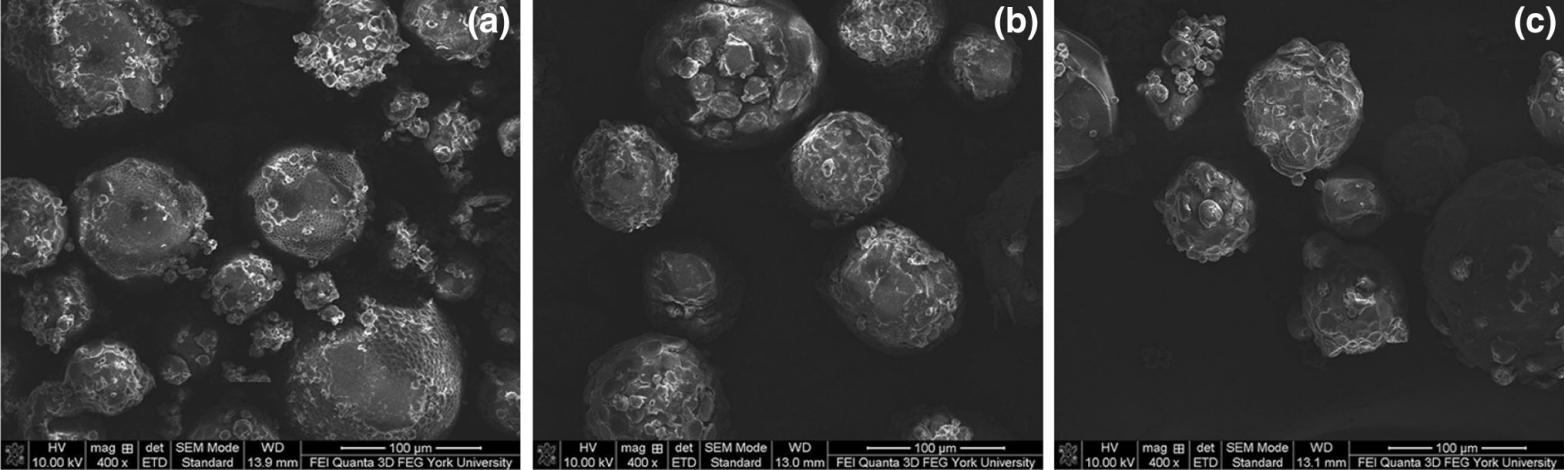
SEM micrographs of PLA–PA microPCM that consist of different core contents: **a** PCM0.4; **b** PCM0.6; and **c** PCM0.8

Figure 4a–c illustrates SEM micrographs of microPCM consisting of 0.4, 0.6, and 0.8 g of PA while keeping a fixed PLA content (i.e., 1.2 g). It can be observed that the microPCM’s shapes and surface morphologies were virtually unchanged as the PA content increased. Furthermore, while the microPCM demonstrated their sphericity, some uneven surface morphologies with the presence of tiny microspheres were observed.

**Fig. 5:**
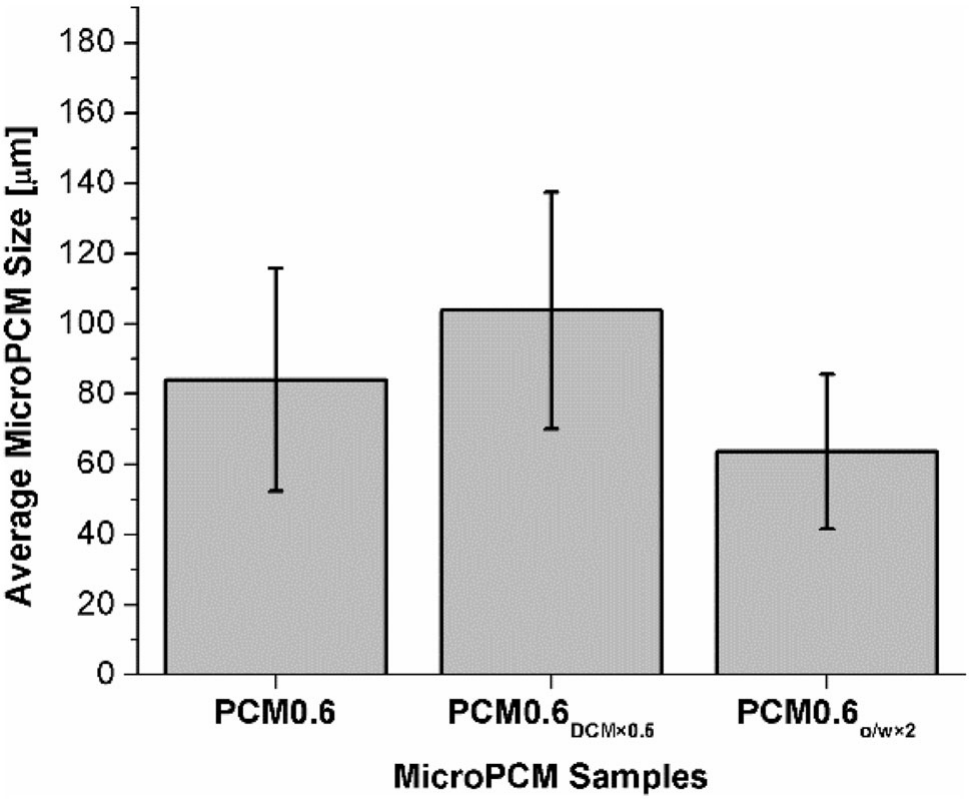
Effects of oil and aqueous media on microPCM’s sizes

**Fig. 6:**
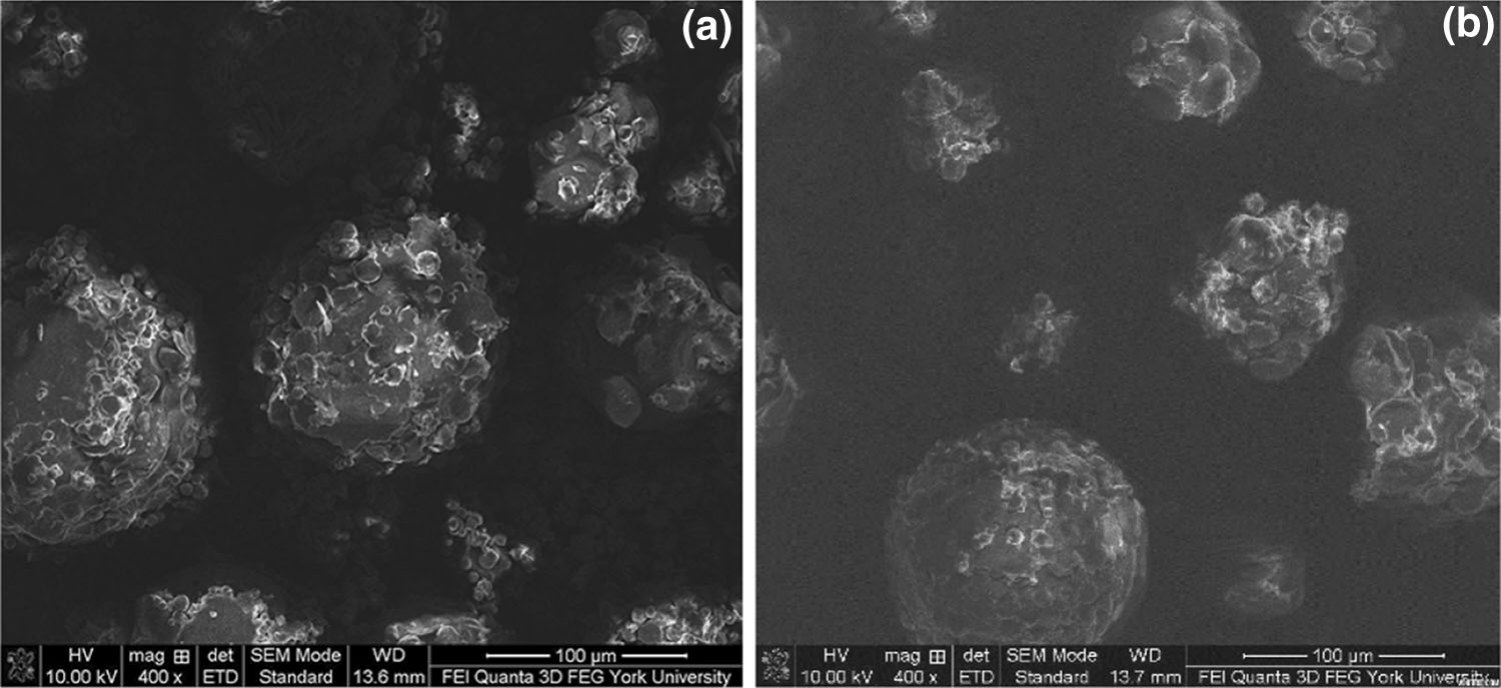
SEM micrographs of PLA–PA microPCM fabricated by different material compositions: **a** PCM0.6_DCM×0.5_ and **b** PCM0.6_O/W×2_

The effects of oil and aqueous media on the microPCM morphologies and sizes were investigated by varying either the amount of DCM or the oil phase-to-aqueous phase ratio. Figure 5 reveals that the average size of microPCM decreased as the oil phase-to-water phase ratio doubled. This can be attributed to the reduced amount of emulsifier (i.e., PVA) in emulsion system, which enhances the ability for the PVA molecules to adsorb on the surface of the dispersed oil phase instead of forming PVA micelles. The governing mechanism of this observation can be found in the later part of this manuscript when discussing the effects of emulsifier content on the microPCM’s sizes and morphologies. Figure 6a shows the SEM micrograph of microPCM prepared by using half the DCM volume of the base case when preparing the oil phase. It can be observed that reducing the DCM content resulted in a higher degree of microPCM agglomerations as well as rougher surfaces. Reducing the amount of organic solvent used would increase the viscosity of the dispersed phase, and thereby lead to larger microPCM sizes as shown in Fig. 6. This trend was again consistent with the prediction based on Eq. (2). By comparing Fig. 6b to Fig. 4b, it can be observed that the sphericity and surface morphology of microPCM were not affected by changing the oil phase-to-aqueous phase ratio from 1:12 to 1:6 in the emulsion.

**Fig. 7:**
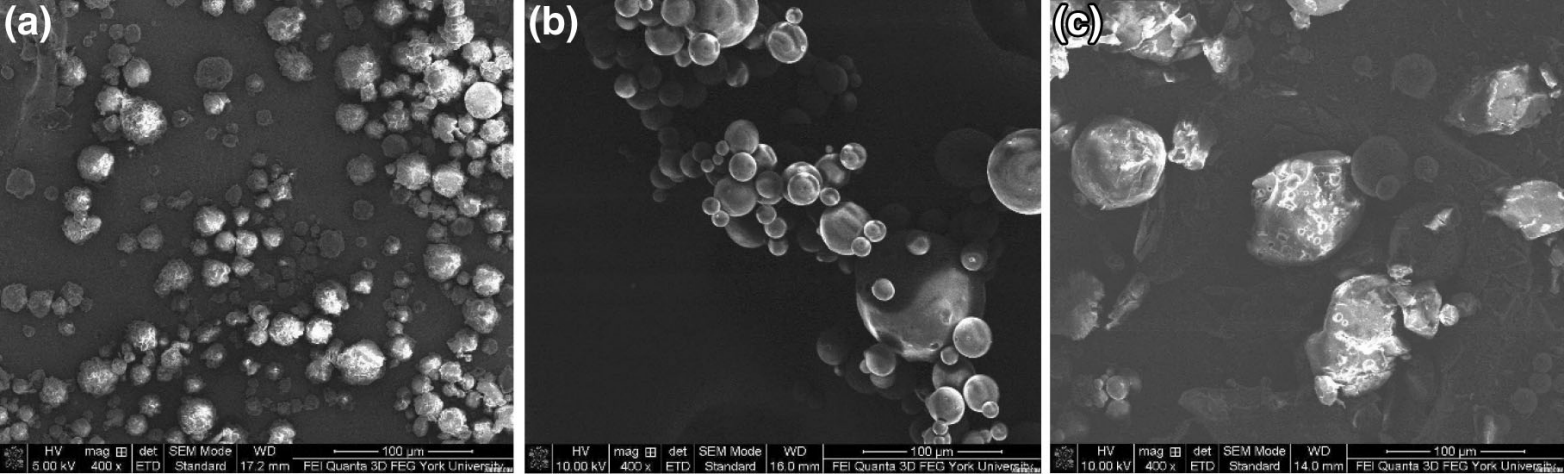
SEM micrographs of PLA–PA microPCM fabricated by different material compositions: **a** PCM0.6_PVA3_; **b** PCM0.6_SDS3_; and **c** PCM0.6_SDS0.5_

Different loadings of two different emulsifiers (i.e., SDS and PVA) were used to prepare microPCM. Comparing the two types of emulsifier as shown in Fig. 7a–c, the uses of SDS eased the filtration and washing steps during the fabrication process. Unlike the case of using PVA as the emulsifier, no residues of surfactant were seen in the samples prepared by SDS. It is believed that this can be attributed to SDS’s higher HLB and its higher solubility in water.

**Fig. 8:**
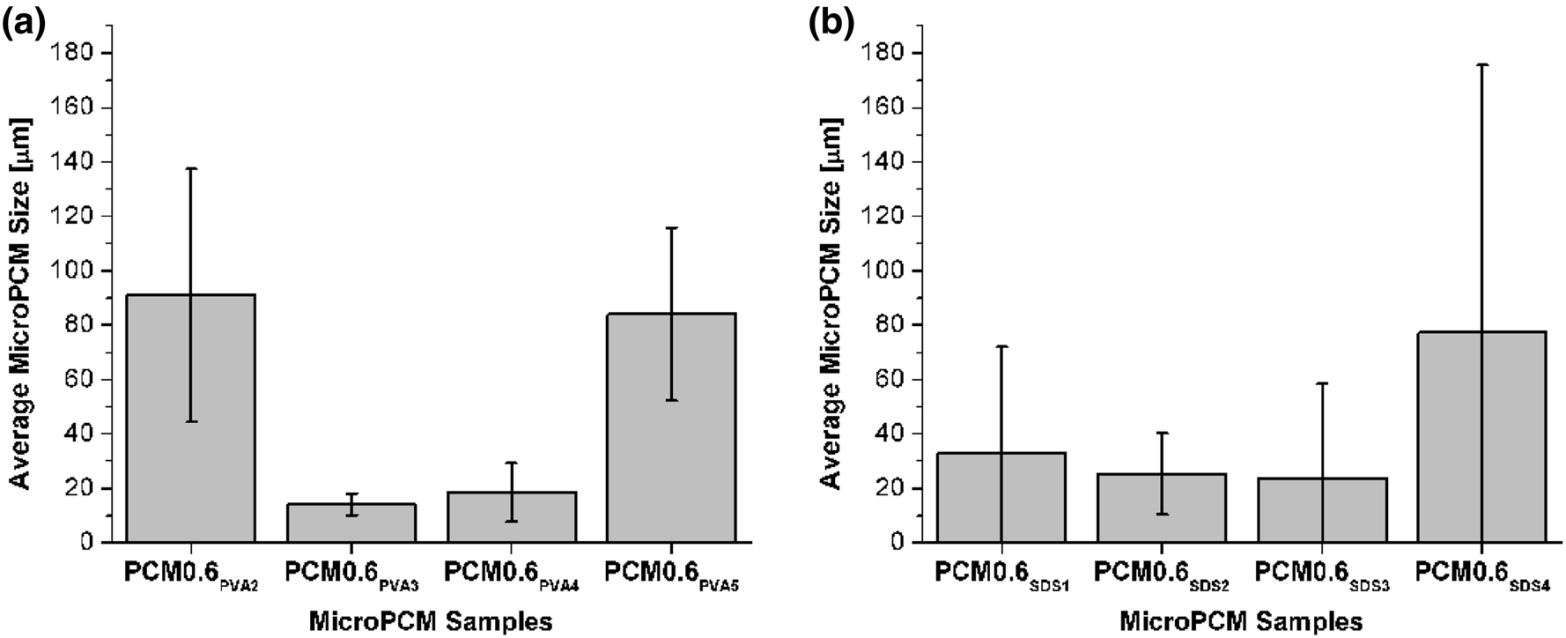
Effects of emulsifier type and content on microPCM’s sizes: **a** PVA and **b** SDS

The effects of emulsifier contents on the microPCM’s surface morphologies and sizes were studied. Figure 8a and b show that, regardless of the type of emulsifier, there existed a U-shaped relationship between the emulsifier content and the average microPCM size. By increasing the amount of emulsifier from a very low content, more surfactant molecules were available to adsorb to the oil–water interface and reduce the surface energy. This would allow the dispersed oil phase to achieve smaller droplet sizes, which also yielded larger total surface area. However, when the emulsifier content continued to increase beyond a threshold concentration, the fabricated microPCM became larger. This threshold concentration is called the critical micelle concentration (CMC) in the oil-in-water emulsion. Over the CMC, excess PVA molecules would more likely form micelles among themselves, rather than adsorbed on the surfaces of the dispersed oil phase [44, 45]. This reduced affinity of the surfactant molecules at the oil–water interfaces would promote the aggregation of dispersed oil phase, and thereby led to the formation of larger microPCM. This was evidenced by the presence of small microspheres (i.e., PVA micelles) on the surfaces of microPCM, as shown in Fig. 4a–c, in all samples prepared by using 5 wt% of PVA solution as the aqueous phase. In contrast, PVA residues were virtually invisible on the surfaces of the microcapsules and there were negligible agglomerates in Fig. 4b and 6a. This could be attributed to the lower number of PVA molecules caused by the reduced concentration of PVA or decreased amount of aqueous phase. Furthermore, it is also interesting to note that, for microPCM prepared using SDS, reducing the emulsifier content to 0.5 wt% would yielded very small number of microPCM while a large amount of un-encapsulated materials were observed. In other words, the extremely low emulsifier content caused a low encapsulation efficiency of the PA core. As discussed in an earlier section, this low SDS content was investigated in an attempt to overcome the loss of PA to the aqueous phase when higher loading of SDS was used.

## Conclusions

Solvent evaporation method, which was commonly used in the pharmaceutical industry for drug encapsulation, was applied to microencapsulate a bio-based PCM [i.e., palmitic acid (PA)] with a bio-based polymeric shell [i.e., polylactic acid (PLA)]. Successful encapsulation of PA core by PLA shell was confirmed by Fourier transform infrared analyses. Parametric studies were conducted to investigate the effects of core content, organic solvent content, emulsifier type and content, as well as oil phase-to-aqueous phase ratio on the characteristics of PLA–PA microPCM. Experimental data indicated that higher PA content would yield higher core content. Moreover, increasing the PA content or decreasing the DCM content would slightly increase the microPCM's size due to the higher viscosity of the oil phase. Regardless of the type of emulsifier (i.e., PVA and SDS), increasing the emulsifier content below the critical micelles concentration (CMC) would reduce the microPCM size due to the reduction of surface energy at the oil-to-water interface by the surfactant. However, further increase in emulsifier content above this threshold concentration would result in larger microPCM sizes. This can be attributed to the tendency of PVA molecules to form micelles among themselves. Furthermore, while the high hydrophile-lipophile balance of SDS led to smoother microPCM surfaces, its high solubilizing capability also led to the dissolution of PA in the aqueous phase. This resulted in the loss of PA and very poor encapsulation efficiency. Therefore, SDS was not a proper surfactant for PA-PLA microcapsules. In short, successful fabrication of 100% bio-based microPCM is expected to promote further the environmental sustainability of the already environmentally friendly latent heat energy storage technology.
